# Metabolic Versatility of *Mycobacterium tuberculosis* during Infection and Dormancy

**DOI:** 10.3390/metabo11020088

**Published:** 2021-02-02

**Authors:** Dorothy Pei Shan Chang, Xue Li Guan

**Affiliations:** Lee Kong Chian School of Medicine, Nanyang Technological University, 59 Nanyang Drive, Singapore 636921, Singapore; PEISHAND001@e.ntu.edu.sg

**Keywords:** metabolism, *Mycobacterium tuberculosis*, tuberculosis, infection, dormancy, systems biology

## Abstract

*Mycobacterium tuberculosis* (*Mtb*), the causative agent of tuberculosis (TB), is a highly successful intracellular pathogen with the ability to withstand harsh conditions and reside long-term within its host. In the dormant and persistent states, the bacterium tunes its metabolism and is able to resist the actions of antibiotics. One of the main strategies *Mtb* adopts is through its metabolic versatility—it is able to cometabolize a variety of essential nutrients and direct these nutrients simultaneously to multiple metabolic pathways to facilitate the infection of the host. *Mtb* further undergo extensive remodeling of its metabolic pathways in response to stress and dormancy. In recent years, advancement in systems biology and its applications have contributed substantially to a more coherent view on the intricate metabolic networks of *Mtb.* With a more refined appreciation of the roles of metabolism in mycobacterial infection and drug resistance, and the success of drugs targeting metabolism, there is growing interest in further development of anti-TB therapies that target metabolism, including lipid metabolism and oxidative phosphorylation. Here, we will review current knowledge revolving around the versatility of *Mtb* in remodeling its metabolism during infection and dormancy, with a focus on central carbon metabolism and lipid metabolism.

## 1. Introduction

Despite the availability of treatment, *Mycobacterium tuberculosis* (*Mtb*), the causative agent of tuberculosis (TB), remains a highly successful pathogen circulating globally, with almost 10 million new cases and 1.2 million deaths per year [1]. There is a resurgence of TB cases and the spread of multidrug resistant (MDR) TB. The secret to *Mtb*’s success lies in its ability to evade host immune defenses and anti-TB drugs [2], and to persist in the absence of growth. *Mtb* exhibits metabolic flexibility, contributing to its long-term persistence and its ability to cause latent TB in a staggering one-fourth of the human population [3]. These individuals are reservoirs of carriers as the dormant bacterium can potentially reactivate, leading to manifestation of active TB. The patient will develop symptoms including persistent cough and can spread the disease through airborne particles containing *Mtb*.

As *Mtb* navigates through the human host system to establish an infection, it needs to withstand and adapt to various harsh environments, such as varying acidity, osmolarity and nutrient-deficient microenvironment. The metabolic versatility of the tubercle bacilli plays a critical role in its survival and persistence in limiting conditions within the host. As a heterotroph, *Mtb* is capable of metabolizing multiple carbon sources, which can be synthesized by the bacterium or acquired from the host cells [4,5,6,7]. The bacterium is further able to switch between replicative and non-replicative states, through rerouting of its metabolic networks, in response to the varying host environments. Dormancy refers to the reversible non-replicative state in which the tubercle bacilli are viable but exhibit low or minimal metabolic activity (reviewed in [8,9,10,11]). This regulated phenomenon is induced when the bacilli are subjected to unfavorable growth conditions, including nutrient limitation or low oxygen. Once returned to favorable conditions, the metabolic rate of *Mtb* reverts to normal, and growth of the bacilli is reactivated, which can lead to establishment of active infection. Dormant *Mtb*, due to its low or minimal metabolic rate, is able to evade the actions of anti-TB drugs, which target actively growing bacterium. Phenotypic resistance is also observed in another subpopulation of bacilli, known as persisters (reviewed in [12]). These bacilli have consistently low metabolic rates even under optimal growth conditions. The ability of dormant and persister subpopulations of *Mtb* to survive under antibiotic exposure is a major concern in anti-TB treatment as they are difficult to eradicate and further serves as a pool of bacterium, which over a long period of antibiotics exposure can potentially acquire genetic mutations of resistance.

The metabolic capability and versatility of *Mtb* has clearly contributed to its success as a pathogen during establishment of infection and its persistence (reviewed in [13]). Moreover, the susceptibility of *Mtb*, and other pathogens, to antibiotics treatment is clearly dependent on their metabolic states [14]. Indeed, with the renewed appreciation of the critical roles of metabolism in *Mtb* survival during infection and dormancy, development of drugs targeting mycobacterial metabolism is an obvious potential anti-TB strategy. Numerous studies using classical genetics and biochemistry of specific key enzymes have led to the understanding of the relevance of mycobacterial metabolism during infection and dormancy. Since metabolism is an intricate process that involves a vast network of enzymes and metabolites, the emergence of system biology approaches has in recent years allowed us to piece the metabolic jigsaw puzzle together and contributed to a more comprehensive view of the metabolism of mycobacteria.

This review aims to discuss the current knowledge revolving around the versatility of *Mtb* in remodeling its metabolism during (1) infection and (2) stress and dormancy, with a focus on central carbon metabolism and lipid metabolism. We will discuss instances of novel drugs that target mycobacterial metabolism, but this is not exhaustive, and readers are referred to more extensive reviews available [15,16,17]. Finally, we will present how recent applications of systems biology approaches have contributed to a more coherent view of the metabolic responses of *Mtb.*

## 2. *Mycobacterium* and Its Metabolic Versatility during Infection

During the process of infection, *Mtb* transits between airborne droplets, mucosal epithelia, alveolar macrophages, necrotic cells and caseous granulomas [18]. The plasticity of its metabolism enables *Mtb* to adapt to the varying environments that it colonizes. Metabolism of carbon sources plays a large role during infection by *Mtb*. In bacteria, there are two types of growth behavior: diauxic and coutilization of carbon sources. In diauxic growth, the bacterium metabolizes the carbon source that supports the fastest growth. When this carbon source is depleted, the bacterium subsequently metabolizes the next preferred carbon source with the involvement of carbon catabolite repression [19]. *Mtb* on the other hand has a preference for coutilization of carbon sources during its growth [5]. Specifically, it catabolizes multiple carbon sources simultaneously via compartmentalization of discrete metabolic process both in vitro [5] and in macrophages [6,7]. The metabolic versatility is an important adaptation mechanism for *Mtb* to thrive in the nutrient-poor macrophage phagosomes during infection. In this section, we will discuss how *Mtb* metabolizes a range of carbon sources during infection.

### 2.1. Fatty Acids

By consensus, fatty acids, rather than carbohydrates, are proposed to be the primary carbon source for *Mtb* during infection. As early as 1956, Segal and Bloch had demonstrated that *Mtb* from lungs of infected mice preferentially metabolize fatty acids ex vivo [20]. Gene expression analysis further revealed the upregulation of *Mtb* genes involved in fatty acid catabolism during infection [21,22,23]. For instance, *mce1*, which encodes for a fatty acid transporter, and *lucA*, which is necessary for Mce1-mediated fatty acid import, are upregulated during infection [24]. Using a systematic transposon *Mtb* mutant screen, it was further revealed that mutations in the *mce1* locus conferred in vivo growth defects [25]. The requirement of fatty acid transport via Mce1 is further corroborated by fitness defects of Δ*mce1 Mtb* and *M. bovis* bacillus Calmette-Guérin *(M. bovis* BCG) mutants in infected macrophages [26,27] and mice [28,29]. It is noteworthy that a de novo type I fatty acid synthase (FAS-I) was found to be overexpressed in the *mce1* mutant [30], which may serve as a compensatory mechanism for the reduction of fatty acid import by increasing de novo fatty acid synthesis.

**Figure 1:**
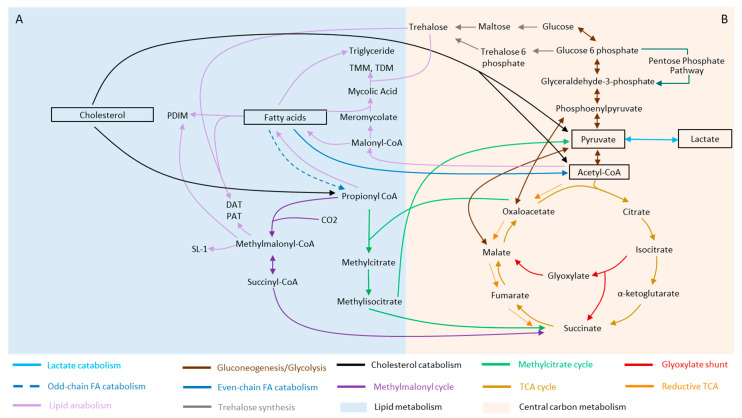
Schematic diagram linking central carbon metabolism (CCM) and lipid metabolism in *Mycobacterium tuberculosis* (*Mtb*). (**A**) Catabolism of cholesterol and fatty acids (dark blue and black arrows) produces a variety of substrates, including succinyl-coenzyme A (CoA), propionyl-CoA and acetyl-CoA, which are channeled into CCM. While acetyl-CoA enters the tricarboxylic acid cycle (TCA) cycle (gold arrows), succinyl-CoA and propionyl-CoA enter the methylmalonyl cycle (dark purple arrows) and methylcitrate cycle (green arrows), respectively. Dashed (- -) dark blue arrow indicates catabolism of odd-chain fatty acids while solid dark blue arrow indicates catabolism of even-chain fatty acids. Fatty acids further serve as building blocks for other lipids, including triglycerides, which are involved in dormancy, while methylmalonyl-CoA serves as precursors of more complex *Mtb* lipids, including sulfolipids, acylated trehalose (light purple arrows). (**B**) *Mtb* is able to adapt to varying conditions through the diversity of its central carbon metabolism pathways, including the pentose phosphate pathway (dark green arrow), and variations in the TCA cycle. When under favorable conditions, carbon will flow through the classic TCA cycle (gold arrows), favoring the biosynthesis of precursors and generation of adenosine triphosphate (ATP). During infection when the bacterium is exposed to various stress conditions, carbon intermediates can also go through the reductive branch of the TCA cycle (orange arrows) or glyoxylate shunt (red arrows) in *Mtb*. These pathways are important for the regeneration of metabolites such as succinate, which is essential for instance in adaptation to hypoxia. During growth, intermediates of the TCA cycle must be withdrawn for the biosynthesis of fatty acids, nucleotide bases and amino acids through gluconeogenesis. These intermediates are replenished to achieve steady levels for normal TCA function by anaplerosis. The anaplerotic node is the metabolic link between glycolysis, gluconeogenesis (dark red arrows) and the TCA cycle (gold arrows) and acts as a switch that directs the flow of carbon distribution within the CCM. An example of anaplerosis is during growth on fatty acids, when the glyoxylate shunt (red arrows) serves to replenish malate via isocitrate lyase and malate synthase. Glucose further serves as a precursor for trehalose (grey arrow), which is a building block of glycolipids. Abbreviations: SL-1 = sulfolipid-1; FA = fatty acid; TCA = tricarboxylic acid cycle, CoA = coenzyme A; CO_2_ = carbon dioxide; DAT = diacyl trehalose; PAT = poly-acyl trehalose; PDIM = phthiocerol dimycocerosate; TMM = trehalose monomycolate; TDM = trehalose dimycolate. Note: the schematic is a simplified form and do not represent all steps of the biosynthesis and catabolic pathways of the various metabolites.

*Mtb* is able to metabolize both even-chain and odd-chain fatty acids (Figure 1A, dark blue arrows). During fatty acid catabolism, even-chain fatty acids are broken down to acetyl-coenzyme A (CoA) (Figure 1A, dark blue arrow, solid line) and utilized by the TCA cycle while odd-chain fatty acids are broken down to propionyl-CoA (Figure 1A, dark blue arrow, dashed line). Subsequently, propionyl-CoA must first be converted into a less toxic form before it can be used in the TCA cycle. There are three main mechanisms developed by *Mtb* to counter and detoxify propionate accumulation for its survival (Figure 1A). The first mechanism occurs via the methylcitrate cycle (MCC) (Figure 1A, green arrows) where isocitrate lyase 1 (Icl1) functions as a methylisocitrate lyase and converts propionyl-CoA to succinate [31,32]. The second mechanism is via the vitamin B12-dependent methylmalonyl pathway (MMP) [33] (Figure 1A, dark purple arrows) where propionyl-CoA is first converted to methylmalonyl-CoA and subsequently transformed to succinyl-CoA. The third mechanism involves the diversion of derivatives of propionyl-CoA, such as methylmalonyl-CoA, into the biosynthesis of methyl-branched fatty acids (Figure 1A, light purple arrows), which can be further incorporated into cell wall lipids, including phthiocerol dimycocerosate (PDIM), sulfolipid (SL) and di-and poly-acyl trehaloses (DAT, PAT) [34].

Fatty acid metabolism is clearly not a standalone by itself. Rather, it is intimately linked to central carbon metabolism via the common intermediate, acetyl-CoA, which is directed into the various metabolic branches, including the TCA cycle (Figure 1B, gold arrows), the glyoxylate shunt (Figure 1B, red arrows) and the gluconeogenic pathway (Figure 1). Phosphoenolpyruvate carboxykinase (PEPCK) is essential for growth of *Mtb* on fatty acids and catalyzes the flow from TCA cycle-derived metabolites to gluconeogenic intermediates, highlighting the potential link between fatty acid metabolism and the gluconeogenic pathway [35] (Figure 1A,B). The involvement of the glyoxylate shunt enzymes, isocitrate lyases (Icl1/Icl2), in utilization of fatty acids and its impact on *Mtb* growth and virulence [36] (Figure 1B,C), further suggested the multitude of mechanisms involved in fatty acid utilization for the survival of the bacterium.

### 2.2. Cholesterol

Cholesterol, another lipid-based carbon source, has been established by various studies as a nutrient for *Mtb*’s growth during infection [4,37,38]. Although *Mtb* does not synthesize cholesterol, encoded in its genome are genes involved in cholesterol metabolism and transport [4]. Acquisition of host cholesterol through the Mce4 transporter system was found to be crucial for persistence in the lungs of chronically infected animals and for growth within the interferon gamma activated macrophages [4]. Early genetic screens predicted that the mce4 genes, encoding for the Mce4 complex that is required for cholesterol import, was required for *Mtb*’s survival when passaged in mice during 2–4 weeks of infection [25]. Lipid uptake coordinator A, LucA, was also found to interact with Mce4 complex to import cholesterol [24], suggesting an overlap between fatty acid and cholesterol uptake during infection.

Several studies have further provided support for the role of host cholesterol in mycobacterial growth and infections. Specifically, *Mtb* genes, *kshB*, *fadA5* and *hsaC*, which are involved in cholesterol metabolism, are required for optimum growth and persistence of *Mtb* in vivo [39,40,41]. Through a chemical screen for inhibitors that target cholesterol metabolism, it was found that cAMP is required for cholesterol utilization by *Mtb* [42]. Interestingly, the group of Platt found that macrophages infected with persistent intracellular *M. bovis* BCG and *Mtb* resembled the phenotype of Niemann Pick Type C cells, including accumulation of cholesterol and sphingolipids in late endosomal and lysosomal compartments [43], which can serve as a potential store of lipids for *Mtb*. Evidently, *Mtb* is able to utilize fatty acids and cholesterol from the host during infections. However, whether these lipids serve to fulfill the same or different metabolic requirements remain a topic for further investigation.

### 2.3. Lactate and Pyruvate

Besides the long-established lipid-based diets, a more recent addition to our current knowledge of potential primary carbon sources for *Mtb* is lactate, a metabolite directly linked to pyruvate. The activity of phosphoenolpyruvate carboxykinase, PckA, is essential for utilization of lactate [44]. Further characterization involving the combination of classical microbiology with a “multi-omics” approach consisting of ribonucleic acid (RNA) sequencing (RNA-seq) transcriptomics, proteomics, stable isotopic labeling coupled with mass spectrometry-based metabolomics, led to the findings that lactate and pyruvate metabolism required both the glyoxylate shunt (Figure 1B, red arrows) and the methylcitrate cycle (Figure 1A, green arrows) [45], which were both previously associated with fatty acid metabolism [31,35,36]. The requirement for pyruvate, however, depends on the lineage within the *Mycobacterium tuberculosis* complex (MTBC), in part arising from mutations of *pykA*, which encodes for pyruvate kinase [46]. While more works are required to further elucidate the contribution of lactate and pyruvate as a major carbon source for *Mtb* during infection and its impact on mycobacterial physiology, the current findings highlighted a common link between fatty acid utilization and metabolism of lactate and pyruvate, which involves the glyoxylate shunt and methylcitrate cycle [44] (Figure 1). This also brings to question whether the previously determined inability to establish infections in Δ*pckA* [35] and Δ*icl1/2* [36] knockout mutants are linked to lactate and pyruvate metabolism, besides lipid metabolism.

Evidently, the metabolism of *Mtb* continues to be a subject of immense interest given its central roles in virulence and growth. Although fatty acids are still believed to be the main carbon source used by the bacterium in vivo and in vitro, it is likely that other nutrients including cholesterol and lactate, and the less well-characterized host-derived sphingomyelin [47] serve to meet distinct metabolic requirements during growth under different conditions. For instance, the acquisition of host cholesterol may play an important role for chronic infection but is not necessary for establishing infection [4]. This implies that during the establishment of infection, other carbon sources play a greater role in supporting the growth of *Mtb*, while cholesterol is involved in counteracting the harsh environment during chronic infection. Indeed, the diet of *Mtb* may be influenced by its site of replication and host cell type. In particular, *Mtb* is able to egress from the phagosome to the cytosol, where it will be exposed to a metabolic milieu distinct from the nutrient limiting phagosomal environment [48,49,50]. Future works involving the determination of metabolite levels in these subcellular components where *Mtb* resides, and labeled metabolite tracing using mass spectrometry or imaging technologies will shed new lights into the precise menu *Mtb* is able to access and utilize for its survival and persistence.

## 3. Remodeling of Metabolism during Stress and Dormancy

As an intracellular bacterium, *Mtb* has evolved strategies to adapt to the varying environments that it colonizes. When exposed to stress conditions, including nutrient starvation [51] and low oxygen [52], *Mtb* is able to switch its metabolism to enter a dormant state while maintaining its adenosine triphosphate (ATP) levels for long term survival within the host [53]. Classical genetics studies have contributed to the identification of factors involved in stress adaptation and dormancy, including DosR, enduring hypoxic response (EHR) and WhiB3, which affect mycobacterial metabolism [54,55,56]. In more recent years, independent studies on dormancy employing a range of systems of biology approaches, including transcriptomics, proteomics and metabolomics analyses, have revealed differential regulation of metabolic networks linked to the electron transport chain (ETC), energy metabolism and lipid metabolism [57,58]. Understanding the essentiality of metabolism during dormancy is critical for the development of more effective therapy for TB. This section consolidates relevant findings on remodeling of lipid and central carbon metabolism of *Mtb* during stress and dormancy.

### 3.1. Rewiring of Carbon Metabolism during Stress and Dormancy

One of the mechanisms by which *Mtb* alters its carbon metabolism in times of stress is by controlling the flux of carbon intermediates through the TCA cycle (Figure 1B, gold arrows). Variations in the TCA cycle in *Mtb* reflect its adaptive capability to diverse metabolic niche and needs [59]. Although the *Mtb* genome is annotated to encode a complete TCA cycle [60], *Mtb* lacks the canonical α-ketoglutarate dehydrogenase (KDH) [61], suggesting a bifurcated TCA cycle. The reductive branch of the TCA cycle (Figure 1B, orange arrows) is, on the other hand, focused on the production of metabolites such as succinate, which is used to sustain membrane potential, ATP synthesis and anaplerosis [62,63,64]. In fact, production and metabolism of succinate is critical for the adaptation of *Mtb* to hypoxia [63]. Alterations of succinate and malate levels through the reductive branch of the TCA cycle was also observed when *Mtb* is exposed to antibiotic-induced stress [65], oxygen depletion [63,66] and high salt concentration [67]. The involvement of the reductive branch of the TCA cycle in mycobacterial adaptation was further supported by upregulation of several enzymes involved in this pathway, including fumarate reductase and phosphoenolpyruvate carboxykinase, during hypoxia. In addition, Icl1, which is involved in the glyoxylate shunt, was also upregulated in hypoxic cultures [57] and antibiotics exposure [65]. Taken together, the enzymes of the reductive branch of the TCA cycle may play an essential role in the adaptation of *Mtb* to different types of stresses. This pathway further serves to maintain an energized membrane under anaerobic conditions, through intracellularly generated fumarate that acts as an electron sink [66]. Moreover, the reduction of fumarate may reoxidize reducing equivalents that could favor cometabolism of several carbon sources under oxygen-limiting conditions [66].

Rerouting of carbon metabolism is also observed upstream of the TCA cycle. Specifically, hypoxia-induced metabolic shift towards the pentose-phosphate pathway (PPP) (Figure 1B, dark green arrow) was observed in both *Mycobacterium bovis* BCG and *Mtb* [68,69]. The PPP serves as a source of reducing equivalents for reductive biosynthesis and the retardation of glycolysis and facilitation of metabolic reprogramming toward the PPP may be associated with achieving a redox balance during stress [69]. This will require further investigations, as the PPP is also a potential source of intermediates for de novo peptidoglycan synthesis, which is reinitiated during recovery from hypoxia [68].

*Mtb* also undergoes metabolic remodeling when under acidic pH conditions, which the bacterium frequently encounters within the mycobacterial vacuole. Interestingly, the ability to grow under low pH is dependent on the carbon sources the bacterium is grown in [70]. It was further demonstrated that the strong induction of genes, including *pckA* and *icl1*, to reroute the distribution of carbon flux through the anaplerotic node is required for promoting growth under acidic conditions. Specifically, PckA connects oxaloacetate to phosphoenolpyruvic acid (PEP) (Figure 1B, dark red arrows), while Icl1 allows the bypassing of the oxidative branch of the TCA cycle and funnel metabolism towards the glyoxylate shunt (Figure 1B, red arrows). The involvement of the anaplerotic node is also evident during nutrient stress, where phosphoenolpyruvate kinase is linked to the production of TCA intermediates from phosphoenolpyruvate [71].

Besides adapting to stress during persistence and dormancy by remodeling central carbon metabolism and rerouting metabolites through variant TCA cycles, *Mtb* can control the flow of carbon metabolites through the trehalose catalytic shift. Trehalose is a non-reducing glucose disaccharide found in *Mtb* and serves as a form of carbon storage [72,73]. It is also a core component of cell surface trehalose monomycolate (TMM) and trehalose dimycolate (TDM). In persister subfractions of in vitro mycobacterial biofilm cultures, trehalose and maltose are diverted from the biosynthesis of cell wall TMM and TDM and are channeled into the biosynthesis of CCM intermediates in order to maintain ATP and antioxidant biosynthetic activities [74]. In addition, the trehalose catalytic shift enables *Mtb* to resist bedaquiline (BDQ)- or carbonyl cyanide m-chlorophenyl hydrazone (CCCP)-mediated side effects and promote transitioning into viable but nonculturable cells (VBNCs) following first-line TB drug treatment [74]. This metabolic shift allows *Mtb* to better adapt to its environment as it ensures sufficient carbon flux into energy production. Interestingly, non-replicating and drug tolerant *Mtb* from hypoxic cultures showed downregulation of TMM and TDM and remodeling of trehalose metabolism [57,68,75]. Furthermore, comparative analysis of clinical drug-resistant and drug-sensitive *Mtb* isolates revealed that the trehalose synthase/amylase (TreS)-mediated trehalose catalytic shift was active in extensively drug resistant (XDR)- and totally drug resistant (TDR)-*Mtb* isolates [74]. Together, these studies highlight the involvement of trehalose metabolism remodeling as a possible adaptive process in harsh environment to ensure sufficient ATP production and may be involved in persistence and drug resistance.

### 3.2. Lipid Metabolism of Mtb and Its Remodeling during Stress and Dormancy

When faced with stress, *Mtb* restricts its growth by diversion of carbon metabolism away from growth-promoting pathways such as the TCA cycle to triacylglycerol (TG) synthesis [76], reinforcing the intimate link between carbon and lipid metabolism in *Mtb*. Cumulating evidence are available demonstrating the extensive remodeling of mycobacterial lipids, including TG accumulation and cell wall thickening, during stress and dormancy [77]. In this section, we will focus on how key *Mtb* lipid classes (based on Lipid Maps classification [78,79])—fatty acyls, glycerolipid, glycerophospholipids and saccharolipids—are modulated during stress and dormancy.

#### 3.2.1. Fatty Acyls (FA)

Besides being utilized by *Mtb* as a carbon source and serving as the building blocks of membrane phospholipids, fatty acids can induce changes in the phenotype of *Mtb*, resulting in dormancy-like traits. Rodriguez et al. developed an in vitro model where *Mtb* grown in even-length long-chain fatty acids as the sole carbon source acquired slow-growth and drug-tolerant phenotypes that are associated with dormancy [80]. Interestingly, treatment of *Mtb* with exogenous unsaturated fatty acids, including arachidonic acid, was shown to resuscitate dormant *Mtb* [81]. Arachidonic acid is found in human cells but not in *Mtb*, and further works are required to elucidate the actual contribution of this host-related lipid in disease reactivation.

Another class of fatty acids that play a significant role in dormancy and reactivation is mycolic acid (MA), which is a unique and structurally complex cell wall lipid present in mycobacteria and closely related species. MA is characterized by a very hydrophobic C_42_ to C_62_ fatty acids with C_22_ to C_26_ α-side chains. MA serves as a precursor for more complex lipids, including TDM and TMM, which together play a significant role in cell wall permeability and regulation of entry of small molecules. Genes involved in mycolic acid synthesis are generally downregulated under hypoxic conditions [82], which is further confirmed at the protein expression level [57]. Using path-seq, Peterson and coworkers revealed that the two mycolic acid desaturases *desA1*/*desA2* are regulated by MadR, which initially promote cell wall remodeling upon in vitro macrophage infection and, subsequently, reduces mycolate biosynthesis upon entering dormancy [83]. Repression of fatty acid metabolism genes and mycolic acid synthesis genes may be beneficial to dormant *Mtb* as mycolic acid synthesis is costly in terms of energy requirements [82]. During reaeration, proteins involved in fatty acid synthesis, including fatty acid synthases I-III, and mycolate and methyl-branched chains biosynthesis were upregulated [57]. Together, these studies demonstrated that the activity of fatty acids and their remodeling play a significant role in dormancy and reactivation.

#### 3.2.2. Glycerolipids (GLs)

GLs can be classified into three subclasses: monoacylglycerides (MG), diacylglycerides (DG) and triacylglycerides (TG). Our knowledge on the functions of TG has been built upon *Mtb* and other *Mycobacterium* species. TG are mainly used as energy sources by *Mtb* and are broken down by lipases to generate FAs during starvation [84]. Remarkably, the *Mtb* genome consists of 24 members in the “Lip” family, which have been annotated as putative esterases or lipases based on the consensus sequence GXSXG characteristic of the α/β hydrolase fold family (reviewed in [85,86]). Amongst these lipases, LipY, which belongs to the hormone-sensitive lipase (HSL) family, is able to break down TG from both host and mycobacterial lipids as shown in *M. bovis* BCG-infected foamy macrophage models [87]. In addition, when *Mtb* and *M. bovis* BCG are exposed to stress, they accumulate TG [88,89,90] and the production of TG diverts carbon metabolism away from growth-promoting pathways, thereby restricting the growth of the bacilli [76]. Accumulation of TG and the presence of intracellular lipid inclusion (ILI) is in fact a common hallmark for dormancy. Strikingly, Garton et al. [91] discovered the presence of lipid body-positive mycobacteria in the sputum of samples. Classically, dormant *Mtb* has been associated with the granuloma and the presence of this non-replicating pool of bacterium in sputum raises the question of how this population of dormant bacterium is transmitted.

As an intracellular bacterium, *Mtb* is capable of synthesizing its own TG and deriving the lipid from its host. TG accumulation can be mediated by the fatty acyl-CoA ligase (FACL), an acyl-CoA synthetase [92], and Tgs1, a triglyceride synthase, which is regulated by the dormancy-induced DosR regulon [93]. Upregulation of mycobacterial genes, such as *dosR*, *hspX*, *icl1*, *tgs1* and *lipY*, was observed in *Mtb* within hypoxic lipid-loaded macrophages along with other *Mtb* genes known to be associated with dormancy and lipid metabolism [93]. Using radioisotope and fluorescent labeling, it was shown that *Mtb* used fatty acids released from host TG for resynthesis of TG within the bacterium. In *M. avium*, it was demonstrated that host TG in very-low-density lipoprotein (VLDL) was important for ILI formation and growth arrest [94]. Upon the removal of VLDL, ILI declined and cell division of *M. avium* resumed. Breakdown of TG is also evident in *M. bovis* BCG during recovery from hypoxia-induced dormancy, which requires the actions of TG lipase [89], and it is proposed that TG serves as a pool of FA for mycobacterial growth. The mobilization and incorporation of host lipids for the formation of ILI, which occurs during mycobacterial dormancy was further demonstrated by a separate study using exogenous labeled TGs stored in host lipid droplets to track the uptake of FAs and host phospholipids into *M. marimum*, [95]. In addition to serving as an energy source during starvation, or during reactivation of dormant bacterium, TG can also act as an electron sink for balancing cellular metabolism based on the environmental conditions [96].

#### 3.2.3. Glycerophospholipids (GPs)

Glycerophospholipids (GPs) are fatty acid diglycerides with a phosphatidyl ester and are major components of the plasma membrane. This membrane lipid class can be further classified into various subclasses including cardiolipin (CL), phophatidylethanolamine (PE), phosphatidylinositol (PI), phosphatidylglycerol (PG), LysoPG and phosphatidylinositol mannoside (PIMs) based on their terminal ester groups or presence of mannoside group(s). GP can serve as potential donors of fatty acids for formation of TG in ILI [95]. However, there are limited studies on the exact roles of GP in dormancy. Nonetheless, modulation of membrane fluidity through alterations of phospholipid composition serves as a protective mechanism for the bacilli when exposed to varying environmental stresses. For instance, when *Mtb* is exposed to physiologic salt concentration, which is generally higher than laboratory culture conditions, plasma membrane associated PE and PG levels decreased [67], while an increase in PIMs with higher acylation state such as Ac_2_PIM_2_ and Ac_2_PIM_6_ was observed [67].

#### 3.2.4. Saccharolipids

Saccharolipids are lipids in which fatty acids are linked directly to a sugar backbone and are found in various organisms besides *Mtb* [97]. Extensive structural diversity exists in saccharolipids due to the variations in both the carbohydrate and lipid moieties. For instance, sulfolipids (SL) and sulphoglycolipids (SGL) possess a sulfur-containing functional group in the sugar moiety. *Mtb* can alter its SL and SGL composition during persistence [70] and dormancy [75]. Transcriptional profiling data of *Mtb*, in both glycerol and pyruvate at pH 5.7, identified strong induction in the *mmpL8-pks2* operon [70], which has been shown to control the synthesis of SL [98]. Using a radiolabeled ^14^C-acetate tracing experiment at pH 7 and pH 5.7, it was demonstrated that accumulation of SL occurred in wildtype *Mtb* but not in Δ*phoPR* mutants [70]. This confirmed the effects of acidic pH in remodeling lipid metabolism through stimulation PhoPR and promotion of SL accumulation. In hypoxia-induced dormancy, the biosynthetic genes for SL, acylated trehalose (PAT/DAT), PDIMs and methylmalonyl are downregulated [75]. Conversely, these genes are induced upon reactivation and reaeration [75], confirming the ability of *Mtb* to remodel saccharolipids and other lipids during its transition towards dormancy or the active state.

Collectively, numerous studies have demonstrated that *Mtb* undergoes extensive metabolic remodeling when under stress and during dormancy. Metabolic remodeling is a survival strategy developed by the bacterium and a detailed understanding of the functions of metabolism under varying conditions can provide novel insights into potential points of interventions to eradicate the persistent and deadly bacterium.

## 4. Metabolism in Drug Discovery

**Figure 2:**
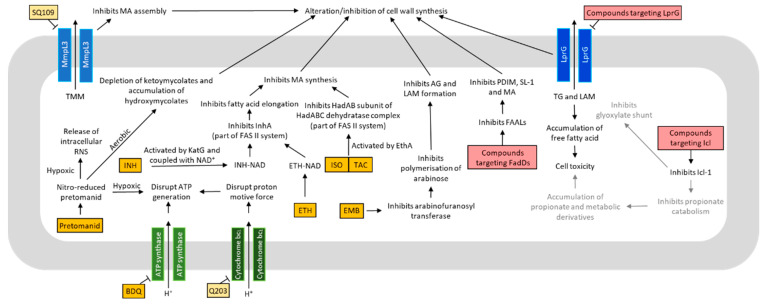
Mechanisms of action of various antituberculosis (anti-TB) drugs and compounds targeting the metabolism of *Mycobacterium tuberculosis* (*Mtb*). Many anti-TB drugs and compounds target different aspects of metabolism, and consequently lead to bacteriostasis or bactericidal effects. Some anti-TB drugs and compounds may share the same cellular targets. Others may act on different targets, which can affect common metabolic pathway(s). Drugs approved for treatment are colored in orange. Compounds undergoing clinical trials are colored in yellow. Compounds still in vitro developmental phase are colored in red. Text colored in grey are the proposed mechanisms. Abbreviations: INH = isoniazid; ETH = ethionamide; ISO = isoxyl; TAC = thioacetazone; BDQ = bedaquiline; FadDs = fatty acid adenylating enzymes; ICL = isocitrate lyase; EMB = ethambutol; ATP = adenosine triphosphate; NAD = nicotinamide adenine dinucleotide; LprG = Lipoarabinomannan carrier protein LprG; EthA = flavin adenine dinucleotide (FAD)-containing monooxygenase; HadAB = β-hydroxyacyl-ACP dehydratase HadAB complex; KatG = catalase-peroxidase; FAS II = type II fatty acid synthase; InhA = enoyl-ACP reductase; RNS = reactive nitrogen species; AG = arabinogalactan; TG = triacylglycerol; LAM = lipoarabinomannan; FAAL = fatty acyl AMP ligase; PDIM = phthiocerol dimycocerosates; SL-1 = sulfolipid-1; MA = mycolic acid.

The critical roles of metabolism in mycobacterial growth, virulence and dormancy, in combination with the differences in metabolic pathways between *Mtb* and humans, make mycobacterial metabolism an attractive target for therapeutic development. With the emergence of drug resistant *Mtb*, there is now an urgent need for novel drugs for treatment of MDR- and XDR-TB. Here, we will summarize a range of antimycobacterial strategy that targets metabolism, including oxidative phosphorylation and lipid metabolism (Figure 2). More extensive reviews on this topic can be found in various publications [17,99,100,101,102].

### 4.1. Oxidative Phosphorylation and ATP Production

The central energy currency among all bacteria species, ATP, is resynthesized via various mechanisms, which differ greatly between differentorganisms. For example, the enterobacteria regenerate nicotinamide adenine dinucleotide (NAD) pool by fermentation when grown on carbohydrates and bypasses the need of oxidative phosphorylation [103,104]. *Mtb*, on the other hand, is devoid of fermentative lactate dehydrogenase, making oxidative phosphorylation strictly necessary for growth [105]. In fact, during dormancy, although the bacterium is in general under a state of reduced metabolism, it continues to synthesize ATP, albeit at lowered levels, for its long-term survival. Hence, the oxidative phosphorylation pathway serves as a promising drug target for both replicating and non-replicating *Mtb*. Indeed, bedaquiline (BDQ), a drug that targets the F_0_F_1_ ATP synthase [106,107], was approved by the FDA in 2012 for the treatment of MDR-TB [108]. Critically, BDQ is active against dormant *Mtb* [109]. With increased recognition of drugs targeting the electron transport chain (ETC) as being effective against MDR- and XDR-*Mtb* [53], other enzymes involved in oxidative phosphorylation have gained interest as potential drug targets against *Mtb* [110,111,112,113,114,115]. One such potential drug target is the cytochrome bc_1_ complex [114,115]. Q203, an inhibitor of the cytochrome bc_1_ complex that has completed a Phase 2 clinical trial and is in the pipeline for further development [116].

As research in drug development against *Mtb* advances, it is also important to appreciate the influence of different carbon substrates on the TCA cycle and oxidative phosphorylation and ultimately drug efficacy. This can be exemplified by the actions of BDQ, which is enhanced when *Mtb* is grown on non-fermentable energy sources, including lipids [117]. Interestingly, Greenwood et al. showed that BDQ accumulated primarily in host cell lipid droplets, which served as a reservoir for the transfer of BDQ when utilizing host lipids [118]. Alterations of host lipids in turn affected the efficacy of BDQ against intracellular *Mtb*, highlighting the potential of host cell lipid droplets as an effective delivery system for improving efficacy of anti-TB drugs. The link between carbon sources and drug efficacy is further evident in Q203, which acts through inhibition of cytochrome bc_1_ activity and consequently ATP synthesis [119]. When supplemented with glycerol, it was discovered that *Mtb* significantly upregulated its Cyt-bd terminal oxidase, hence providing an alternative respiratory route and diminishes the effectiveness of cytochrome bc_1_ inhibitors such as Q203 [120]. As glycerol is commonly used in standard laboratory mycobacterial culture media, this research finding highlights the need for more attention in defining suitable conditions for drug screening. The impetus for research on this topic is further exacerbated by the inappropriate use of rich bacterial broth in studies that seeks to mimic the physiology of the site of infection of *Mtb*, which on the contrary is nutrient limiting. This has in fact been one of the most cited reasons for the failure of drug discovery programs [121] and establishment of in vitro conditions representative of in vivo infection and dormancy is critical for TB drug discovery.

### 4.2. Lipid Metabolism

Targeting mycobacterial lipid metabolism as a point of therapeutic intervention dates back to as early as the 1950s, with the discovery of isoniazid (INH) [122,123]. INH, which targets the biosynthesis of MA [124], the major component of mycobacterial cell wall, continues to be one of the first line drugs used for TB treatment. Newer drugs that disrupt MA metabolism, albeit through different mechanisms, were approved over the last 50 years. These drugs include ethionamide (ETH) [125], isoxyl (ISO) [126] and thioacetazone (TAC) [127]. Alternative targets of lipid metabolism include biosynthesis or degradation of fatty acids and metabolites, including fatty acid adenylating enzymes (FadDs) [128,129] and isocitrate lyases. Inhibition of Icl has been well-studied with several known inhibitors including 3-nitropropionate, 3-bromopyruvate [130], phthalazines [131,132], hydrazones [133] and 5-nitro-2,6-dioxohexahydro-4-pyrimidinecarboxamides [134]. However, these are currently experimental compounds and have not advanced into any clinical trials and more research is certainly required to optimize potential leads.

Other than targeting the biosynthesis of lipids or conversion into energy, the transport of lipids into and out of *Mtb* can also be an attractive point of intervention. One such drug target is the trehalose monomycolate exporter MmpL3, which is embedded on the inner membrane and acts as a cytoplasmic membrane transporter for TMM to the cell wall [135,136]. SQ109, a 1,2-diamine related to ethambutol, has completed a Phase II study involving drug-sensitive TB patients in 2016 [137]. Though its mode of action remains unclear, studies have demonstrated that SQ109 targets MmpL3, which directly inhibits TDM production. This in turn leads to the failure of attaching mycolates to arabinogalactan and interference with cell wall assembly [135]. Another transporter for lipids that is a drug target of interest is the lipoarabinomannan carrier protein LprG, which is involved in exporting triacylated lipids, such as TG and lipoglycans, such as lipoarabinomannan, to the mycomembrane [138,139]. In vivo studies have shown that LprG is essential for *Mtb* to establish infection [138], highlighting its potential as a drug target for treatment of infection by multidrug resistant *Mtb.*

Despite the overwhelming need for novel anti-TB drugs, drug development in general is full of challenges, risks and failures. In fact, an earlier study by the group of David Russell involving a screen of compounds with anti-TB effects had clearly demonstrated stark differences in potency of compounds when using laboratory culture media containing glucose and oleic acid, in comparison to *Mtb*-infected macrophages [42]. This further reinforced the confounding factor we raised, that is, the appropriateness of the carbon sources utilized in in vitro screens, which can influence drug efficacy [117,119]. With the improved understanding of mycobacterial metabolism during the host–pathogen interaction, better designs of drug screening assays can be established for discovery new therapeutic regimes for TB.

## 5. Systems Biology Methodology and Novel Applications in *Mtb* Research

There is no doubt that the metabolic versatility of *Mtb* is critical for infection and is one of the mechanisms it adopts to overcome the actions of antibiotics and stress. With the advent of omics technologies, we are in a better position to obtain a more coherent view on mycobacterial metabolism [6,57,58,75], including understanding the (1) intimate links between lipid and central carbon metabolic networks, (2) interactions between two organisms, the human host and the bacterium and (3) heterogeneity of bacterial population. The latter is greatly facilitated by the recent boom in single-cell technology. In this section, we will discuss more recent advances and applications of systems biology approaches to delineate complex networks during *Mtb* infection and dormancy.

Infection involves the intimate interactions of the host and the pathogen, and advancement in transcriptomics, specifically development of dual-RNA sequencing, has enabled the analysis of both the pathogen and host cells from infected samples. Application of dual-RNA sequencing has recently led to the discovery of a divergent transcriptional response of *Mtb* when comparing infection within alveolar macrophages and interstitial macrophages [140]. *Mtb* growth is sustained in alveolar macrophages where the bacilli have increased access to iron and fatty acids, while in interstitial macrophages, *Mtb* growth is restricted due to a high level of nitric oxide and iron sequestration. These findings emphasize the significance of the cell-type dependent metabolic interface in supporting *Mtb* growth in permissive host macrophages and restricting *Mtb* growth in nonpermissive host cells. Path-seq, which was developed more recently to overcome the sensitivity limits of dual-RNA sequencing, enables the analysis of minute amounts of *Mtb* transcripts within up to million-fold excess host RNA [83]. Path-seq analysis of *Mtb* infection using an animal model revealed a novel transcriptional program for mycobacterial cell wall remodeling when *Mtb* infects alveolar macrophages in mice [83]. Specifically, the mycolic acid desaturase regulator, MadR, was found to be involved in the transcriptional modulation of mycolic acid desaturases, which in turn led to cell wall remodeling during initial infection and entry into dormancy. These technologies are clearly extremely powerful for studies of intracellular *Mtb*, which factors in the effects of the host environment, in contrast to in vitro models.

The integration of multiple omics technologies further contributes to a comprehensive view of the transcriptional and metabolic networks of *Mtb* infection and dormancy. Vrieling and coworkers demonstrated with a combination of untargeted and targeted metabolomics approach that *Mtb* infection resulted in elevated intracellular levels of NAD, creatine, creatine phosphate and glutathione compared to uninfected macrophages [141]. From metabolomics alone, it would be impossible to conclude whether changes in these metabolites are due to changes in the enzymatic level or transcriptional regulation level. Within the same study, the authors further performed RNA-sequencing which revealed the altered gene expression of the key metabolic enzyme involved in NAD, creatine, glucose and glutamine metabolism in *Mtb*-infected M2 macrophages [141]. Combining both transcriptomics and metabolomics, it can clearly be concluded that *Mtb* infection modulates host macrophage metabolic pathways and the link between the exact genes involved in the observed metabolome changes can be established. The metabolic networks of *Mtb* and infected host cells were established by Zimmermann and coworkers through a combination of dual-RNA sequencing and metabolic flux balance analysis. In this integrated omics study, the research group revealed that *Mtb* can coutilize up to 33 different nutrients during early macrophage infections [6]. These metabolites are funneled to specific processes, with three predicted for biomass production, and the rest for energy production and precursor formation. Given that metabolism is a rapid process, we propose that lipidome- and/or metabolome-wide ^13^C flux analysis can be further applied to capture dynamic features in real time, including the rate of synthesis, interconversion and degradation of lipids and metabolites [7,142] during infection and dormancy. Besides the derivation of novel mechanistic insights from -omics based approaches, transcriptome and metabolome-based markers can serve as potential markers for prediction of TB progression [143,144].

One of the bottlenecks of systems scale analyses of metabolites and lipids are the chemical diversity of these biomolecules, which limits the coverage of the metabolome and lipidome. The applications of lipidomics and metabolomics in mycobacterial research is increasingly common [6,145,146] and in fact databases for *Mtb* lipids were previously established to facilitate identification of mycobacterial lipids [79,147]. To expand on the coverage of metabolomes, a multiplatform approach can be undertaken, for instance by using nuclear magnetic resonance and mass spectrometry, or to employ distinct separation techniques, including chromatography or ion mobility, to capture metabolite subsets, which vary in their specific physiochemical properties. Such a combinatorial approach allows higher levels of metabolite coverage, sensitivity and specificity as compared to the conventional single platform approach [148]. Fernández-García et al. combined time-of-flight (ToF) mass spectrometry coupled to capillary electrophoresis, gas chromatography and liquid chromatography, to capture the global metabolome changes in the lungs of *Mtb*-infected mice. This enabled the identification of various host-associated metabolic pathways linked to the progression of TB in the mouse model [149]. Metabolomics can be further applied to studies of mechanisms of actions of previously uncharacterized antimicrobial compounds [150], characterization of vaccine candidates [151] and have potential in drug discovery programs of *Mtb* [152].

Another exciting development, which can have significant impact on mycobacterial research is the advances in single-cell technologies. Within an infection, the bacterial population exhibits phenotypic variations, including survival, clearance, persistence and host death, which suggest that heterogeneity of cellular behavior [153,154]. Single-cell based technology when coupled with omics approaches can offer a higher resolution to decipher the heterogeneity within bacterial population. Maglica, Özdemir and McKinney developed a combinatorial method comprising of high-throughput microfluidics and automated microscopy to explore single-cell tracking of intracellular ATP in live *M. smegmatis*. This approach has advanced studies of bacterial energy metabolism in response to drug treatment at an individual cell level [155,156]. Specifically, it was discovered that *M. smegmatis* persisters are able divide in the presence of INH and exist in a dynamic state of balanced division and death due to stochastic pulses in KatG expression and consequently INH activation [156]. While single-cell tracking has contributed to substantial insights into mycobacterial metabolism during drug responses and persistence, the combination of the technology with omics approaches is technically more challenging. The use of single-cell transcriptomics on *Mtb* has met little success due to the trace amounts of transcripts below detection levels. Nonetheless, the implementation of single-cell transcriptomics on host cells have led to novel insights in interindividual variations in responses to TB infection [157] and identification of markers to discriminate between healthy controls to latent TB infection and active TB [158]. Future advances in the technology to capture the transcriptome and metabolome of *Mtb* at the single cell level will certainly expand our knowledge on the physiology of this deadly pathogen.

Our discussion on the metabolic versatility and diversity of *Mtb* will not be complete without acknowledging the natural genetic and metabolic variations of *Mtb* isolates circulating in the world. Population genomics of *Mtb* has provided strong evidence of the genotypic differences between *M. tuberculosis* complex (MTBC), which is classified into seven lineages based on their genomes [159]. Interestingly, within lineage 2, the hypervirulent Beijing sublineage was shown to accumulate TG, as a result of constitutively upregulation of DosR dormancy regulon [160,161]. Lipidomics and metabolomics analyses of MTBC isolates further confirmed the metabolic diversity of MTBC [162,163]. To unravel the genetic basis for the metabolic diversity of MTBC, the group of Gagneux developed an approach for integration of genomics and metabolomics data to identify mutations that are associated with strain-specific metabolic adaptive capacity and vulnerability [162]. The integrative omics approach has also led to the discovery of the genetic basis of baseline differences in susceptibility to the antibiotic *para*-aminosalicylic acid. Collectively, the applications of systems biology technologies and their integration are extremely powerful for research on TB, which involves the complex interactions of the bacterium with its host and environment.

One of the promising aspects with the increased data generation using omics technologies is the possibility to integrate data from the same experimental model(s), which is produced by different approaches and/or performed by independent research groups. The sharing of these omics data can hence make a major contribution to the field of integrative omics as research groups can capitalize on published data that is made publicly available to derive deeper insights into the biology of *Mtb*. For instance, to investigate how the cyclic adenosine 3′,5′-monophosphate (cAMP) receptor protein (CRP) is linked to downstream metabolic changes, Liu et al. performed metabolomics and stable isotope tracing and compared the metabolome of wild type *Mtb* and *crp* deletion mutant [164]. Their data suggested the regulatory roles of CRP in nitrogen metabolism and peptidoglycan synthesis, which was corroborated by mining a previously published transcriptomics data [165]. This is made possible with data sharing through various platforms, including the National Centre of Biotechnology Information (NCBI)′s GEO Datasets [166,167], BioCyc’s *Mtb* pathway/genome databases [168], the Pathosystems Resource Integration Center (PATRIC) [169], the MycoBrowser [170] and European Bioinformatics Institute’s Metabolights [171]. However, data sharing remains a major bottleneck due to the lack of standardization of experimental models and data structure and should be handled with caution. The problems arising from integration of -omics data from different research groups has been addressed in a review by Furness [172]. Nonetheless, a recent initiative has been made by the group of Gagneux, to define a reference set of genetically well-characterized MTBC isolates, spanning all seven known human-adapted MTBC [173]. This MTBC clinical strain reference set can be used by any research groups to study the impact of phylogenetic differences on phenotypes and physiology of MTBC. With the efforts towards harmonization through the clinical strain collection and data collection using various technologies, we anticipated that such systematic and detailed analyses will improve our understanding of TB and contribute to the development of new approaches to control the disease.

## 6. Conclusions

*Mtb* can metabolize multiple carbon sources simultaneously and adapt its metabolism to support its growth and survival under varying conditions. While it is known that *Mtb* favors the usage of host lipids in vivo, new lines of evidence suggest alternative carbon sources, including lactate, pyruvate and sphingolipids, as potential requirements for its growth and virulence. The metabolic versatility of *Mtb* has also contributed to the bacilli’s ability to persist and remain dormant in host macrophages, a stage that the bacterium exhibits low metabolic rates and is phenotypically resistant to antibiotics. Dynamic metabolic remodeling is also involved in the promotion of growth during resuscitation of *Mtb* from the non-replicative state. Hence, it is paramount to understand the metabolism of *Mtb*, given its implications on infection, antibiotics efficacy and potential in novel therapeutics development. This review also discussed recent advances in systems biology approaches and its applications in unraveling the complexity of the metabolic networks involved in infection and bacterial physiology. With the growing availability of large-scale datasets, a more concerted effort in the scientific community to share data will facilitate the integration of the biological networks of *Mtb* to understand the complexity of metabolism during host–pathogen interactions and to unravel insights into *Mtb* biology. A more refined understanding of the metabolism of *Mtb*, which takes into consideration its in vivo conditions and its natural genetic and phenotypic variations, is instrumental for future research. Collectively, a consolidated effort involving systems biology research of MTBC clinical isolates can facilitate identification of more effective targets to overcome infections by this genetically and phenotypically diverse pathogen.
